# Spatiotemporal 3D image registration for mesoscale studies of brain development

**DOI:** 10.1038/s41598-022-06871-8

**Published:** 2022-03-07

**Authors:** Sergey Shuvaev, Alexander Lazutkin, Roman Kiryanov, Konstantin Anokhin, Grigori Enikolopov, Alexei A. Koulakov

**Affiliations:** 1 Cold Spring Harbor Laboratory, Cold Spring Harbor, NY USA; 2 Moscow Institute of Physics and Technology, Moscow, Russian Federation; 3 Center for Developmental Genetics and Department of Anesthesiology, Stony Brook University, Stony Brook, NY USA; 4 Institute of Higher Nervous Activity and Neurophysiology RAS, Moscow, Russian Federation; 5 P. K. Anokhin Institute of Normal Physiology, Moscow, Russian Federation; 6 Lomonosov Moscow State University, Moscow, Russian Federation

**Keywords:** Development of the nervous system, 3-D reconstruction, Image processing, Software

## Abstract

Comparison of brain samples representing different developmental stages often necessitates registering the samples to common coordinates. Although the available software tools are successful in registering 3D images of adult brains, registration of perinatal brains remains challenging due to rapid growth-dependent morphological changes and variations in developmental pace between animals. To address these challenges, we introduce CORGI (Customizable Object Registration for Groups of Images), an algorithm for the registration of perinatal brains. First, we optimized image preprocessing to increase the algorithm’s sensitivity to mismatches in registered images. Second, we developed an attention-gated simulated annealing procedure capable of focusing on the differences between perinatal brains. Third, we applied classical multidimensional scaling (CMDS) to align (“synchronize”) brain samples in time, accounting for individual development paces. We tested CORGI on 28 samples of whole-mounted perinatal mouse brains (P0–P9) and compared its accuracy with other registration algorithms. Our algorithm offers a runtime of several minutes per brain on a laptop and automates such brain registration tasks as mapping brain data to atlases, comparing experimental groups, and monitoring brain development dynamics.

## Introduction

The development of algorithms for transforming similar images into common coordinates (*registration* algorithms) was initially driven by the need to register low-resolution medical images, such as fMRI data^1^. Since then, substantial progress has been made leading to the development of optimized image registration software that is now used in clinical practice. The emergence of whole-brain staining and imaging methods has yielded whole-brain datasets with a single-cell resolution, which require high levels of registration precision (reviewed by Susaki and Ueda^2^). Large datasets containing whole-brain imaging data collected by 3D microscopy labs require high-throughput and precise automatic registration to brain atlases. In this work, we build upon two key existing image registration approaches to produce a set of image registration algorithms that could enable mesoscale-level studies of brain development.

The two main approaches to image registration are *feature-based* and *free-form* registration. *Feature-based registration* allows users to specify pairs of landmarks in two images, which are used in alignment^3^. With increasing the number of features, this procedure can reach user-defined levels of precision. Overall, feature-based registration is a flexible and computationally tractable approach^1^, however, it may require significant input from users. To increase the throughput of feature-based registration, multiple research groups have worked towards automatic feature extraction methods. One successful approach involves searching for features in the frequency domain. Fourier transformations^4^ and wavelets^5^ were used to identify independent image components, which were then registered separately. Feature-based registration techniques have proven to be efficient on low-resolution medical images^6^.

*Free-form methods* use a different, complementary concept of image registration^7^. Instead of aligning hand-picked features, free-form registration methods optimize spatial transformation to maximize the similarity between two images. Following Sederberg and Parry^7^, one may conceptualize free-form registration as deforming a brain sample together with the transparent agarose cube in which it is embedded to increase similarity integrated over the entire image. The similarity between samples is usually evaluated using measures such as correlation, mutual information, or L^2^ norm of differences; as a result, the outcomes of registration depend on data preprocessing^8^. In successful applications, alignment is typically preceded by image smoothing^9^ and involves converting raster images to vector fields^10^.

Free-form registration methods gained popularity due to their ease of automation. Residual discrepancies between samples, inevitably arising from the methods’ insensitivity to small displacements^11^ are shown to not affect major brain regions^9^. These recent free-form registration algorithms are largely efficient and are widely used for mapping high-resolution brain data to brain atlases.

**Figure 1:**
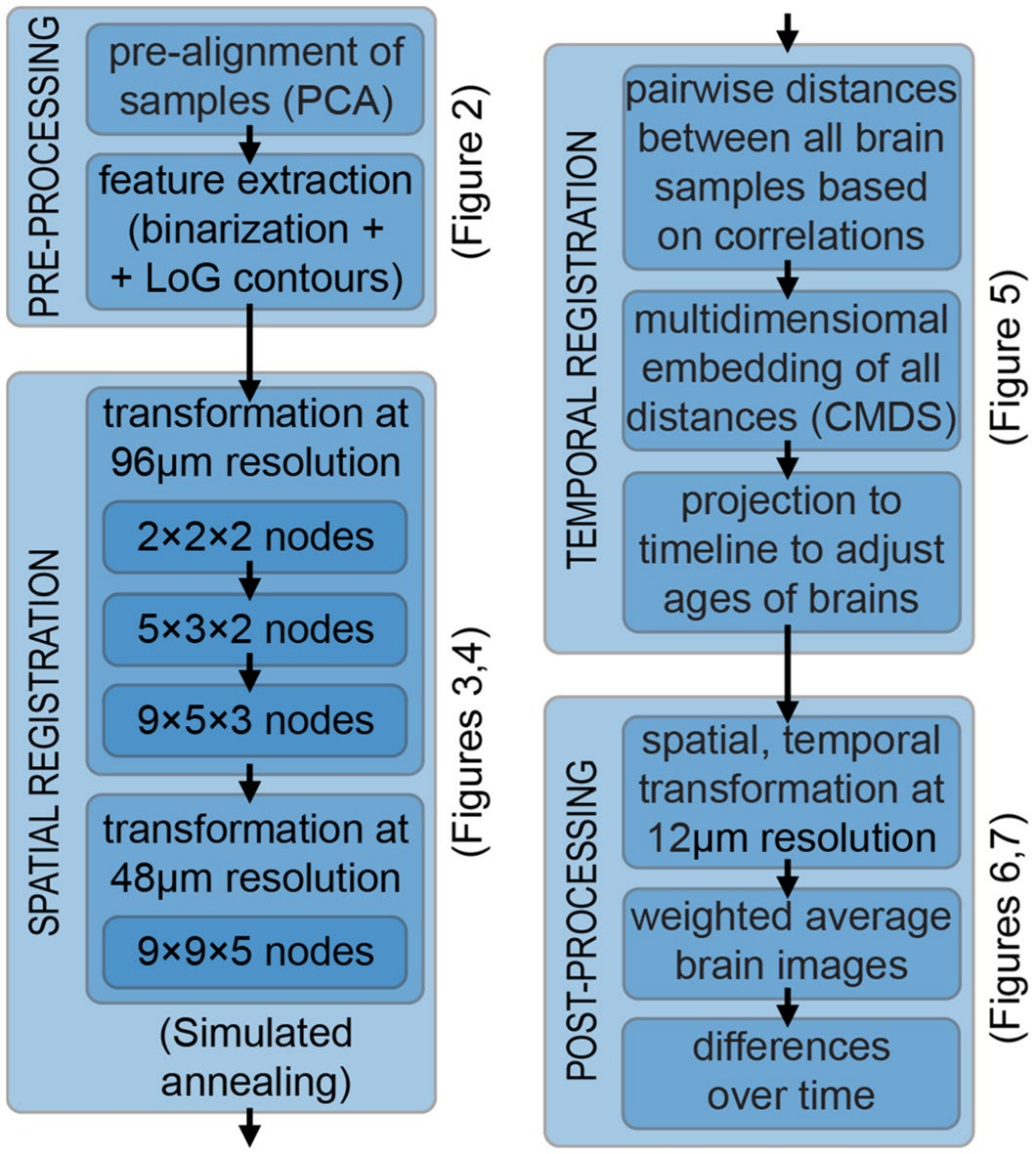
Flow chart of our algorithm for uncovering mesoscale developmental dynamics in the brain. To register brain samples, we start with preprocessing which includes pre-alignment using the principal component analysis (PCA) and extracting the image features to be registered. These features include brain region contours (extracted with Laplacian of Gaussian (LoG) filter) and the overall brain shapes (represented using the binary mask). We then perform spatial registration of brain samples using piece-wise linear deformations and an attention-gated simulated annealing Monte Carlo algorithm. We further register brain samples in time by building a multidimensional embedding where distances between brains are defined by their pairwise dissimilarities (classical multidimensional scaling; CMDS). We project the axis of maximum variance on the timeline, thus obtaining the adjusted ages for each brain sample. We finally display the results by combining the registered brain images with the weights reflecting their adjusted ages. We also compute differential images reflecting short-term developmental dynamics and color-code identified differences.

In this work, we sought to combine the automation of free-form approaches with the precision of feature-based methods to align multiple brains across different developmental stages and to reconstruct mesoscale developmental dynamics of the perinatal mouse brain. We have determined what image features are critical for proper and efficient brain registration and used those in a free-form transformation routine. We introduced routines for estimating the real developmental age for individual samples and for detecting changes occurring in the samples over time (Fig. 1). We applied our procedures to 28 samples of perinatal mouse brains dated P0–P9. We show that our algorithms address challenges specific to the registration of perinatal mouse brains.

## Methods

### Sample data and infrastructure

We illustrated our algorithm using 3D images of 28 whole-mount perinatal mouse brain samples (P0–P9) labeled with 5-ethynyl-2′-deoxyuridine (EdU) to reveal dividing cells^12^ and imaged with a custom Olympus MVX10-based light-sheet microscope^13^ at the Z-resolution of 12 µm and the XY-resolution of 4 µm. To evaluate our algorithm, we used a personal computer (Dell Precision 7510 using 7 GB of RAM and 1 CPU core at 3.3 GHz; the runtime was under 5 min per brain).

### Data import

**Figure 2:**
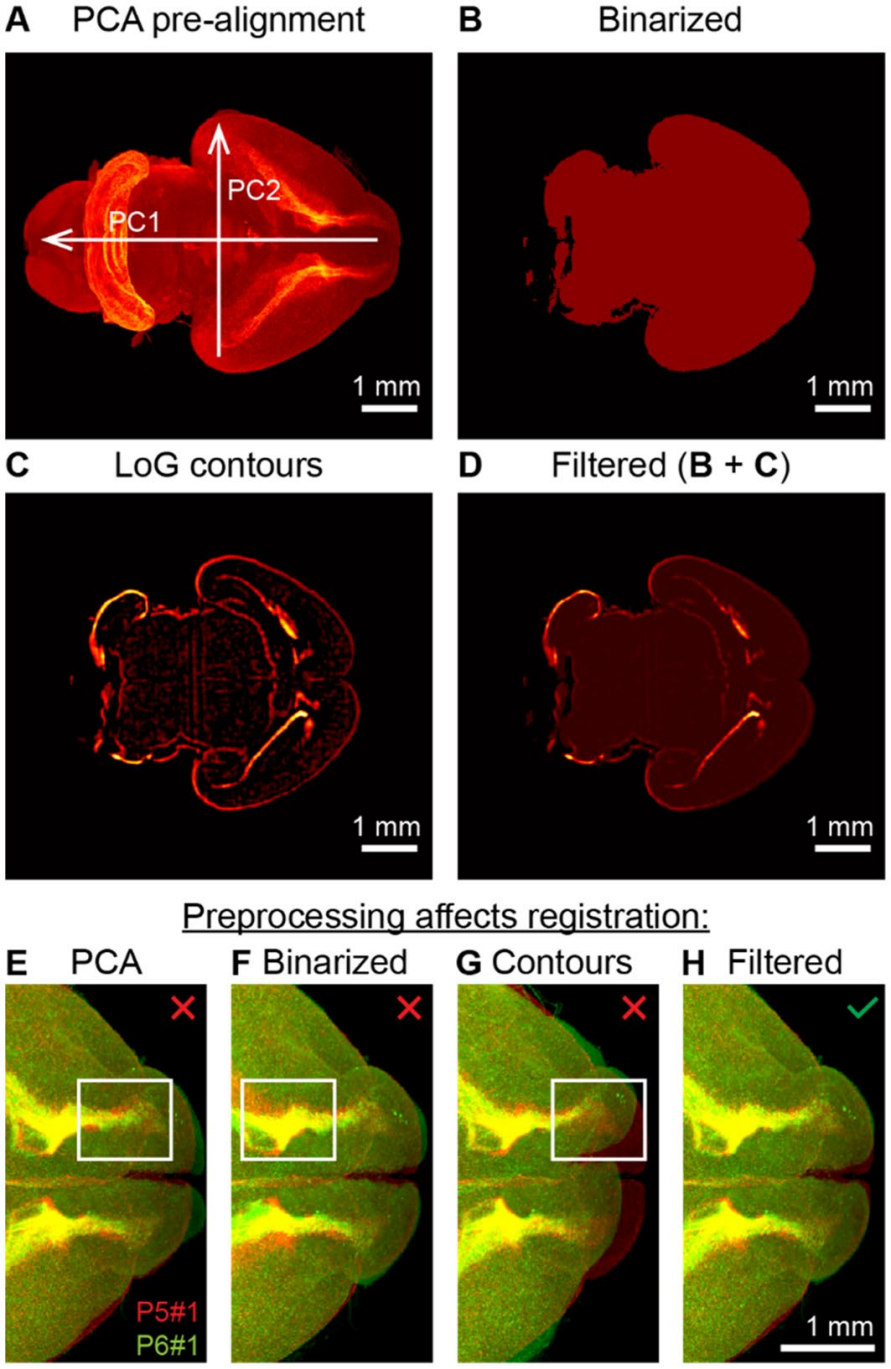
3D image preprocessing steps improving brain registration. (**A**) Brain sample pre-alignment via rotation and scaling using Principal Component Analysis (PCA). (**B**) Cross-section of the binary mask of the brain sample. (**C**) Contours of the brain regions extracted with Laplacian of Gaussian (LoG) filter. (**D**) Filtered image; a weighted sum of (**B**) and (**C**). (**E**–**H**) Registration of two brains taken at P5 and P6 using individual image preprocessing methods as indicated. Registration of raw images (**E**) results in slight mismatches between Rostral Migratory Streams (RMSs). Registration of binary masks (**F**) results in a mismatch of the inner structure. Registration of brain region contours (**G**) may not converge and results in large mismatches between samples. The combination of binary mask and contours (**H**) solves the problems of (**E**–**G**) yielding a higher quality registration.

We downsampled the 3D images of the brains to a uniform resolution of 12 × 12 × 12 µm per voxel. We applied histogram equalization within the XY-slices of these images to equalize the signal intensity along the Z-axis in the samples. To remove the background signal, we selected a threshold to best separate the signal inside and outside the sample (~ 0.01 of the maximum signal). We suppressed the signal below this threshold. To pre-align the 3D images, we used the principal component analysis (PCA)^14^ (Fig. 2A) on binarized versions of the images (1 for voxels > 0.01 of maximum intensity, Fig. 2B). We chose an orientation of a sample along the PCA axes manually based on correlations between binarized samples.

### Feature extraction

We extracted contours of brain regions (Fig. 2C) using the Laplacian of Gaussian (LoG) filter^15^. In the LoG filter, we set the blur radius large enough to average out individual cells in the samples, yet small enough to preserve the shapes of brain regions. We found that the blur radius of 5 voxels provided a good compromise for our images. To accelerate computation, we performed the LoG filtering in the frequency domain. We normalized the intensity of voxels in the contour images (produced with the LoG filter) to have an average value of 1 over non-zero voxels (Fig. 2C). We added the normalized contour images to the binarized images (Fig. 2B) to obtain the filtered images (Fig. 2D).

### Spatial registration

**Figure 3:**
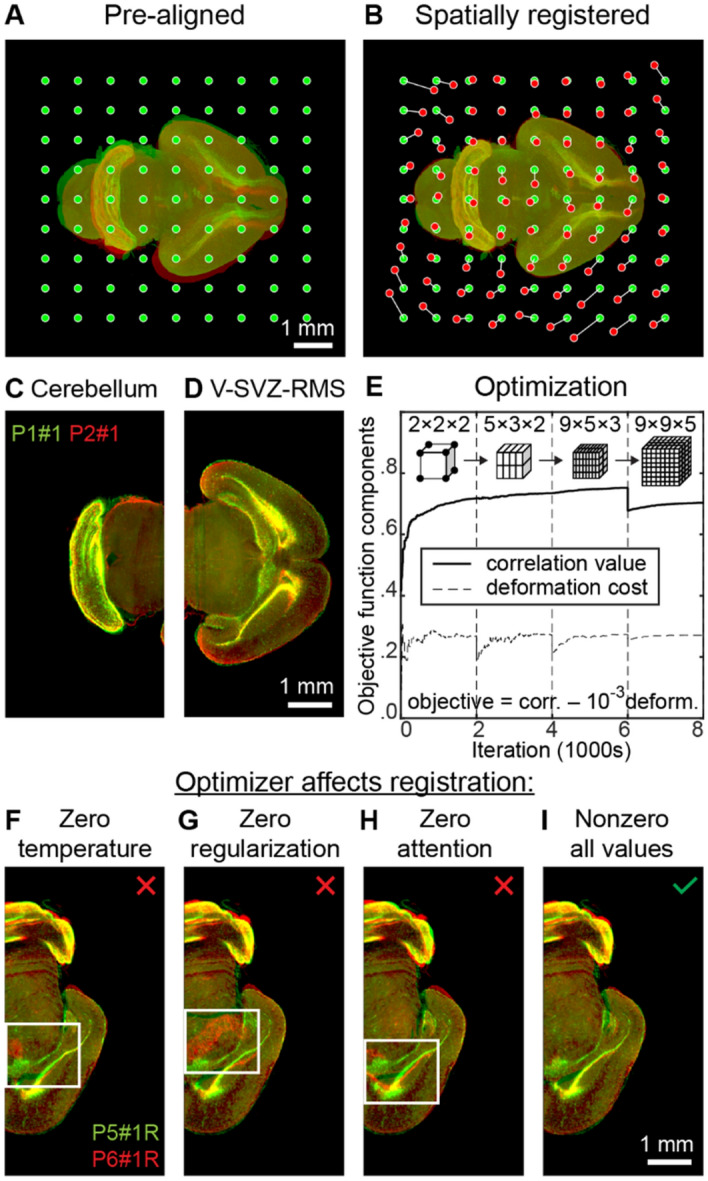
The simulated annealing algorithm allows registering dissimilar developing brains. (**A**) Two brain samples before alignment and the nodes of transformation grid. (**B**) Registered brain samples and displacements of the nodes of the transformation grid. (**C**) Registered cerebellum and (**D**) ventricular-subventricular zone (V-SVZ) and rostral migratory stream (RMS). (**E**) Pearson correlation (similarity) between two filtered brain images (solid line) and deformation cost for the registered brain (dashed line) throughout the simulated annealing registration. The objective function maximized with simulated annealing is a weighted difference between correlation value and deformation cost (1:1000). Simulated annealing is performed in 4 stages, separated by vertical dashed lines, corresponding to different numbers of cells in the transformation grid (2 × 2 × 2; 5 × 3 × 2; 9 × 5 × 3; 9 × 9 × 5) and different image resolutions, defined by linear voxel size (96 µm; 96 µm; 96 µm; 48 µm). The drop of similarity at iteration 6000 is due to refinement of the voxel size (96 µm → 48 µm). (**F**–**I**) Registration of P5 and P6 brains with different optimizers. (**F**) Registration using a greedy algorithm may result in mismatches of some brain structures. (**G**) Registration with unregularized simulated annealing may diverge, resulting in large-scale discrepancies between source and target 3D images. (**H**) Simulated annealing without attention gating, i.e. choosing nodes with equal probability, converges slower than (**I**) simulated annealing with attention gating, i.e. prioritizing nodes with poorly aligned surroundings.

For each 3D image of a brain sample, we defined a set of spatial transformations ranging from coarse to fine. Each transformation was defined by displacements of nodes in a regular grid spanning the entire 3D image; grid spacing specified the coarseness of transformation (Fig. 3A). Displacements of individual voxels in the 3D image were computed via linear interpolation of the grid node displacements (Fig. 3B). To transfer signal intensity from original to new coordinates we used linear interpolation^16^ of signal intensities. We optimized the node displacements to maximize the objective function defined as the Pearson correlation of filtered (Fig. 2D) brain images minus 1/1000 of deformation energy. To estimate the deformation energy, we triangulated the transformed 3D image using the grid nodes, then computed the L^1^ norm of the volume changes in the triangulation compartments.

### Optimization

To optimize the 3D image transformation, we attempted to displace nodes of the transformation grid one by one using the Simulated Annealing (SA) algorithm^17^ (Fig. 3E). We sampled the magnitude of each attempted displacement from a zero-mean Gaussian distribution. The probability for a displacement to be accepted was defined by the equation $p = \min (\exp (\Delta E/t^{o} ),1)$ which depended on a displacement-related change in the cost function, $\Delta E$, and on *temperature* $t^{o}$^17^.

### Optimization procedure

To accelerate the convergence of the algorithm, we were selecting the nodes to be displaced with probability proportional to the L^1^ difference between the source and target images, both filtered, within the cells of the transformation grid adjacent to the node. The coarseness of alignment was defined by the spacing of the transformation grid. The image resolution and the number of nodes in the transformation grid were refined in 4 stages of alignment, ranging from coarse to fine (Table 1; Fig. 3E).

**Table 1:** Stages of spatial alignment.

Stage	I (coarse)	II	III	IV (fine)
Iterations	2000	2000	2000	2000
# of nodes	2 × 2 × 2	5 × 3 × 2	9 × 5 × 3	9 × 9 × 5
Resolution	96 µm	96 µm	96 µm	48 µm
Initial t^o^	10^–3^	10^–3^	10^–4^	10^–5^
Final t^o^	10^–3^/30	10^–3^/30	10^–4^/30	10^–5^/30

We updated the grid spacing at the beginning of each stage of alignment by adding new nodes to the transformation grid in accordance with Table 1. We defined the displacements for newly added grid nodes using linear interpolation of the existing displacements and set the magnitude of the average attempted displacement equal to 20% of the new grid spacing.

At the beginning of each stage of alignment, we also initialized temperatures to be used in determining the acceptance of displacements (Table 1). We were updating these parameters throughout each stage of alignment. Temperatures t^o^ were decreased exponentially; their values at the end of a stage equaled to 1/30 of their values at the beginning of the same stage. The magnitude of the average attempted displacement evolved depending on the success of previous displacement attempts. Specifically, we divided/multiplied the magnitude of the average attempted displacement by 0.99 at each successful/unsuccessful SA iteration. Each stage of alignment was also characterized by the resolution to which we downsampled the 3D brain images on that stage. We defined resolution by the linear size of a voxel, the same for all 3 dimensions (Table 1). Upon completion of all stages of alignment, we applied final transformations, obtained on downsampled images and defined by displacements of the grid nodes, to full resolution images (Fig. 3C,D).

### Temporal registration

To provide additional validation of our approach, we applied our procedures independently to different hemispheres of the same brain. To this end, we split each 3D image of a brain into two separate images of hemispheres, which doubled the number of samples in our dataset. To split an image of a brain close to symmetrically, we used the following procedure. First, we aligned each brain to its mirror image within the volume. This yielded a transformation T. Second, we transformed the original brain using ½ of this transformation, i.e. T/2. This procedure placed the plane of the brain’s symmetry to the middle plane of the 3D volume. Finally, we split the brain into two hemispheres using the middle of the 3D volume as a separatrix. This procedure yielded two images of hemispheres per brain sample.

Then we aligned the right hemispheres of different brains to each other. To set the order of this alignment, we grouped brain samples by age. In each group, we randomly selected a *reference* right hemisphere. We registered the reference right hemispheres to each other in order of age, i.e. P1 → P0; P2 → transformed P1, etc. We further registered the remaining, non-reference right hemispheres to transformed reference hemispheres of the same age. We copied all transformations to the mirror reflected images of the left hemispheres. Thus, we put all hemispheres in the dataset to common coordinates.

Based on these registrations, we computed a distance matrix reflecting pairwise differences between *all* hemispheres in our dataset (correlation distances between *filtered* images; Fig. 5A). We used this distance matrix in the CMDS dimensionality reduction algorithm^18^ to compute the adjusted ages of each hemisphere in the dataset based on its developmental stage (Fig. 5B). Specifically, for each hemisphere, we computed a coordinate along the 1st CMDS dimension. We used these CMDS coordinates in linear regression to estimate the adjusted ages of hemispheres (Fig. 5B). We checked whether the differences between the adjusted ages of two hemispheres within the same brain are small (Fig. 5D).

### Display

To reconstruct the dynamics of brain development, we generated an average-case brain image for every time point within the span of the samples’ *adjusted ages* (Fig. 6D) as follows.

The weight (contribution) of a given brain hemisphere to a given time point was defined by a Gaussian curve with the maximum at the *adjusted age* of the hemisphere and the standard deviation equal to 1/2 day (1/2 of the age step between the groups). At every time point, we normalized the sum of the weights of all hemispheres to one (Fig. 6C). We used these normalized weights to combine (sum) all images of the hemispheres at every time point.

As the normalized weights changed over time, the contributions of different brains to different time points varied forming a 3D animation of transitions between average-case developmental stages. Although our data were registered to a single hemisphere, we appended the animation with its mirror reflection to display the (symmetric) dynamics in the whole brain. To display the brain growth, we performed the linear fit of the brain sample sizes (Fig. 6A,B) and scaled the average-case images accordingly. To reconstruct the differences over time, for every time point we computed a differential image between the average-case images at the current time point and at the current time point minus one day. We color-coded the positive/negative changes in the intensity using red/blue colors respectively (Fig. 6E).

### Baselines and benchmarks

To evaluate the impact of our algorithm’s design choices on brain alignment, we have additionally executed our algorithm with altered parameters as described below. To assess how *preprocessing* affects registration, we prepared a pair of brain samples (P5 and P6) in four different ways: (i) raw images; (ii) binarized images; (iii) contour images; and (iv) a combination of contour and binarized images (see “Feature extraction” section above for the description of the preprocessing procedures). In all four cases, we then have performed the alignment with our algorithm as usual.

To assess how the *optimizer parameters* affect registration, we have aligned the same pair of brain samples (P5 and P6) preprocessed as usual (a combination of contour and binarized images) under four different settings of optimizer: (i) zero temperature; (ii) zero regularization coefficient; (iii) no attention; and (iv) normal settings (non-zero temperature, regularization, and attention; see “Optimization procedure” section above for the description of the optimizer parameters). In all four cases, the non-altered parameters were set to their default values described above.

To compare the registration quality of our algorithm to that of the other registration packages, we performed registration of 27 pairs of neighboring brains in our dataset (sorted by age) using: (i) CORGI (our algorithm); (ii) ClearMAP^19^; (iii) CUBIC^20^. In CORGI (our algorithm), we used twice the usual number of iterations (because we were aligning whole brains, not hemibrains) and a low regularization coefficient (1/100,000 because here we needed a better alignment, not the preservation of features). For ClearMAP and CUBIC, we performed 2 rounds of alignment: (i) using the default parameters and (ii) with 1/32 of the default number of iterations to match the runtime of our algorithm. For these alignments, we used 32 cores on a Supermicro computer in CentOS 8 environment. The software was downloaded via the following links:

- CORGI: https://github.com/KoulakovLab/Registration
- ClearMAP: https://github.com/ChristophKirst/ClearMap2
- CUBIC: https://github.com/lsb-riken/CUBIC-informatics

To evaluate the registration quality, we exported the aligned brain images to ImageJ FIJI where we used them to form RGB images (red = fixed image; green = moving image). To enhance the image contrast, we used the auto-brightness/contrast tool based on optical slices in the cerebellum, the same for fixed and moving images. We then randomly ordered and anonymized these paired aligned 3D images (including all ages and software packages except ClearMAP with the reduced number of steps, as it did not converge). For each image, an expert made *binary* decisions (yes/no) about whether the alignment is satisfactory in five separate brain regions: (i) the lateral edges of the cerebellum (CB edge); (ii) the bulk of the cerebellum (CB bulk); (iii) the rostral migratory stream (RMS); (iv) the subventricular zone (SVZ); and (v) the olfactory bulbs (OBs). The alignment was considered satisfactory if most of the regions’ volume was matching. We then deanonymized the data and computed the percentage of satisfactory alignment for these five brain regions (edge/bulk of cerebellum, RMS, SVZ, OBs) for all four methods of alignment (CORGI, ClearMAP, and CUBIC with default/reduced number of steps). For each algorithm, we also computed the alignment score defined as an average of the percentages defined above.

To assess whether the *temporal alignment* accounts for the variability in the data, we compared day-to-day variability between the average brains in two cases: (i) with no temporal alignment (the brain ages were defined in the experiment) and (ii) with temporal alignment (see “Temporal registration” section above for details), where for each day P1-P2 we computed the weighted average brain images based on the adjusted ages. To estimate the day-to-day variability, we computed the L^2^ norms of the differences between the *filtered* brain images in pairs of consecutive days, then computed an average of these values for each setting (with/without temporal registration).

### Ethical approval and animal care

All manipulations with mice were carried out *in compliance with* the Guide for the Care and Use of Laboratory Animals by the National Institute of Health and with the Russian Federation Ministry of Health Law 267 of June 19, 2003. All methods for animal care and experimental protocols were *approved* by the P.K. Anokhin Institute of Normal Physiology Committee for Care and Use of Laboratory Animals (Protocol 1 of 3/9/2005). This study was carried out *in compliance with* the ARRIVE guidelines.

## Results

The focus of this work was on designing algorithms for reconstructing the developmental dynamics of the perinatal mouse brain via the registration of brain samples in space and time. Several software packages are available for spatial registration of brain samples. NiftyReg package^21^ developed for and used by the fMRI community and its neuroscience-oriented derivatives such as the aMAP^9^ offer state-of-the-art registration for adult mouse brains. Registration of perinatal brains, however, brings new challenges. First, perinatal brains undergo significant transformations in a short time. They grow and change their overall shape as individual brain regions continue to develop. Thus, an algorithm for perinatal brain registration needs to overcome the pronounced shape differences between developing brains. Second, there is uncertainty in what a perinatal brain should look like at an arbitrary moment of development because current brain atlases focus on major stages of development only^22^. Consequently, registration of perinatal brains must keep a balance between the precision of alignment and preservation of the original data. Third, the developmental unfolding of each brain proceeds at its own pace. As a result, algorithms for perinatal brain registration need to compensate for individual development paces by registering the brains in time. Below, we describe our methods to register diverse perinatal brains (Fig. 1). These methods aim to: (i) maximize registration precision in the context of dissimilar brain samples, (ii) preserve the original data to the greatest extent possible, and (iii) account for individual developmental paces. We illustrated each step with examples from 28 whole mount perinatal brain samples (P0–P9) stained against dividing cells with 5-ethynyl-2′-deoxyuridine (EdU^12^).

### Extracting brain region contours yields better registration

High precision of registration is important in studies involving perinatal brains. Brain samples representing distant developmental stages may be largely dissimilar, i.e. they have only a small number of common landmarks, resulting in unreliable registration. Conversely, brain samples representing adjacent developmental stages may be similar, and thus may be reliably registered in sequence. Because alignment errors accumulate over time, it is important to maintain high precision of registration. On the mesoscale level, a precise registration implies the perfect match of individual anatomical structures. In brain atlases, the anatomical structures are delineated using tissue borders^22^ in accordance with morphological staining^23^ or autofluorescence^9^. Thus, the requirement for precise mesoscopic-level registration can be reformulated as a necessity to match the *contours* of anatomical structures.

Aligning the contours of anatomical structures, besides being our immediate objective, also facilitates precise registration. For both raw images and structure contours, the similarity between two brain images is maximized when two brains are perfectly aligned. In the case of a slight displacement, raw images would still retain a massive overlap, keeping similarity almost unchanged. In contrast, displaced structure contours overlap little, making two images less correlated. We, therefore, expect contour-based registration to be more sensitive to structure mismatches when compared to conventional raw-image-based approaches^24^. The same logic, however, predicts difficulty in finding the initial coarse alignment in contour-based registration. Because the contours of unaligned brains are weakly correlated, the problem of aligning them becomes highly non-convex—and consequently much more difficult to solve.

To facilitate the initial coarse alignment while retaining the benefits of a subsequent fine alignment, one may consider registration of the brain area contours combined with the binary mask of the sample (Fig. 2D).

**Figure 4:**
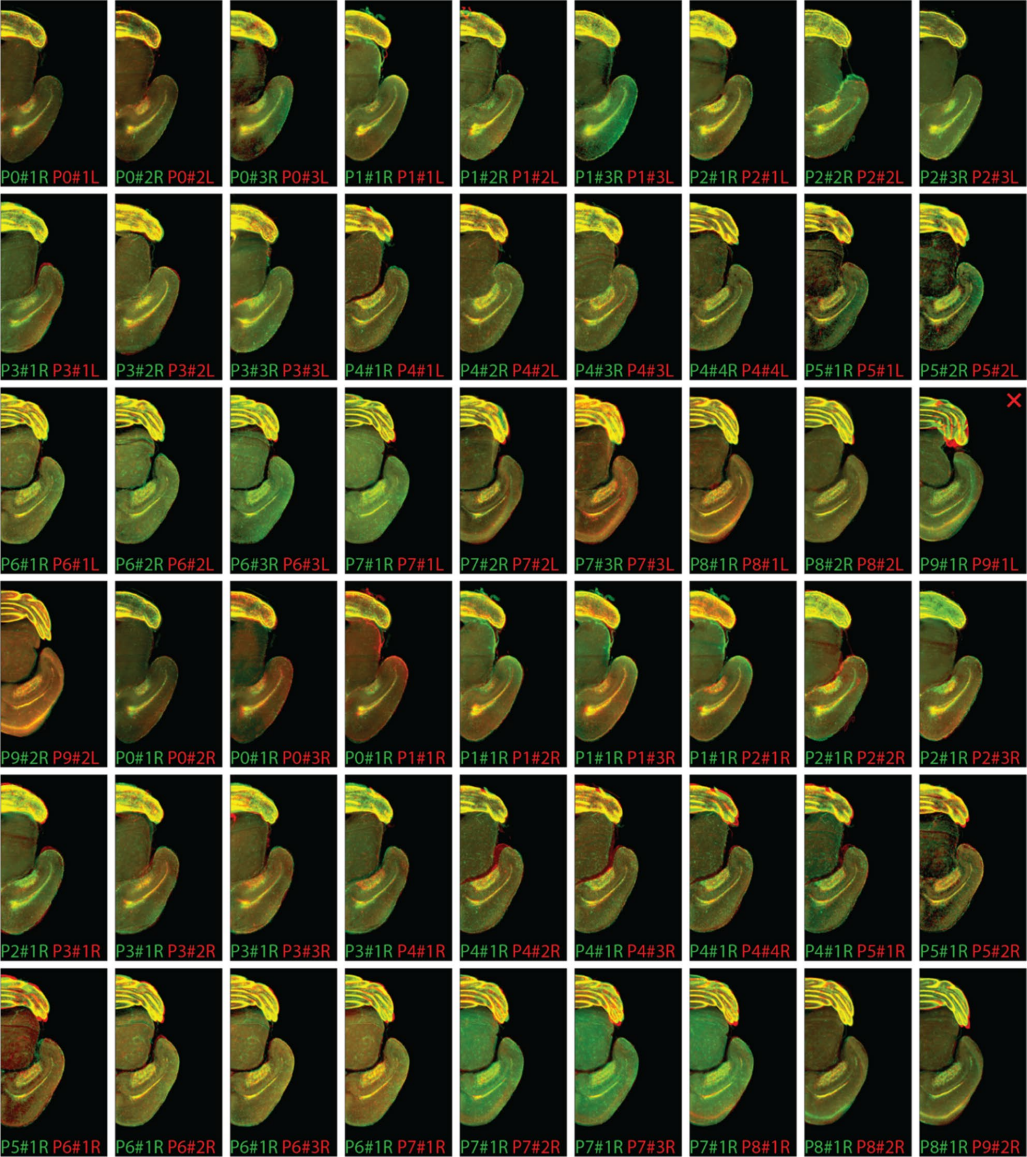
Registration was successful for 53 of 54 pairs of hemispheres from 28 whole-mount samples of developing brains in the testing set. Top three rows and the first image in the fourth row: 28 pairs of left/right hemispheres from the same brains aligned to each other (27 successful). Bottom three rows, beginning from the second image: 26 pairs of pre-registered hemispheres from 27 different brains (the 28th brain with misaligned left/right hemispheres was not used here). For each aligned pair, we show a representative 2D optical section to illustrate the match of individual brain regions and highlight the potential differences. The 1 unsuccessfully registered pair of hemispheres is marked with the “X” sign (see cerebellum).

To test these arguments, we performed registration of a pair of different perinatal brains using raw images (Fig. 2E), brain region contours (Fig. 2G), and a combination of the brain region contours with the binarized images obtained after thresholding the raw images using 1% of maximum intensity as a threshold (filtered image, Fig. 2H). To extract contours of morphologically defined structures in perinatal brains (Fig. 2C), we used the Laplacian of Gaussian (LoG) filter^15^. We also performed registration using the binarized images (Fig. 2F) for completeness. As expected, the raw-image-based registration resulted in an adequate alignment, although some of the brain areas were misaligned (Fig. 2E). The misalignment, despite being relatively small, was impactful in areas such as the olfactory bulb (OB). In the particular example of P5-P6 brains shown in Fig. 2E, the image intensity was dominated by the cerebellum (see Fig. 4), and therefore the Ventricular-Subventricular Zone (V-SVZ) and the Rostral Migratory Stream (RMS) regions had a relatively small impact on the correlation between the source and the target brain images. Moreover, the OBs were mechanically flexible relative to the rest of the brain and, therefore, were distorted during chemical treatments of the samples. Mechanical flexibility paired with the low impact on the overall correlation between samples made the OBs an error-prone region in brain registration and, thus, required additional processing of raw images.

Based on these considerations, in this work, we used an image preprocessing step which combined extracted brain region contours with the binary masks of the samples. Registration performed on such preprocessed data resulted in better alignment of the 3D brain images (Fig. 2H). Specifically, in two aligned images we observed significant overlap between the OBs and RMSs (Fig. 2H). Both components of preprocessing were necessary: in separate *binary-mask* and *contour-based registrations* we observed large mismatches between resulting images (Fig. 2F,G). Overall, a sum of the binarized image and the contours extracted using the LoG filter yielded the best alignment quality among the tested approaches.

### Attention-gated simulated annealing algorithm yields robust registration

Algorithms for brain registration are expected to yield reliable alignment despite variability in samples. This requirement is especially important for perinatal (developing) brains. In the course of development, brain landmarks evolve: the brain grows and changes shape as brain regions continue to be fully formed. Below, we argue that simulated annealing^17^, a Monte Carlo algorithm designed to find optima of non-convex functions, is well-suited to address such variability in samples. We illustrate the algorithm’s performance using 28 samples of perinatal mouse brains.

Pronounced differences in samples are challenging for automatic registration. Many optimization methods used for image registration (e.g. Powell’s, Gauss–Newton, Nelder–Mead, gradient descent, etc.) may require modifications in the conditions of non-convex measures of image similarity^1^. Such modifications include convexifying the task via registering images at different resolutions^1^ or overcoming local similarity minima by varying the optimizer’s step^25^. Such adjustments are necessary because large-scale displacements in brain samples may require correspondingly large steps of the optimizer, but at the same time, larger steps of the optimizer may result in the divergence of an algorithm. In simulated annealing, the problem of non-convex maximization is solved by allowing transient decreases in the objective function (proportional to similarity between the images; Fig. 3E). This way, the algorithm is equipped to escape from local maxima of cost function and to overcome pronounced differences between samples by taking multiple steps which can lead to both its decrease and increase^17^. The algorithm is biased towards an overall increase in the cost function (similarity), which facilitates finding its regional maxima.

Alongside the benefits of simulated annealing, we should consider its potential disadvantages. Monte Carlo methods—the broader class of algorithms—are generally efficient when the number of variables to be optimized does not exceed one hundred^26^. In our setting, the algorithm needs to operate on a grid of up to ~ 400 nodes in 3D space. Nevertheless, this seemingly excessive number of nodes was not a concern in our approach for the following reasons. First, the registration is local, e.g. displacements of the transformation grid nodes in the cerebellum do not interact with the registration of the olfactory bulb. Second, we implemented the attention-gated alignment mechanism that selected the nodes to be altered in proportion with dissimilarity in their neighborhoods (see “Methods”). Finally, at every iteration, we only updated the content of image transformation grid cells adjacent to the displaced node. We, therefore, expected a reasonable convergence rate of simulated annealing in our brain registration task.

We first show that the ability to decrease the objective function is important for brain alignment. To do so, we compared our simulated annealing algorithm (Fig. 3G–I) with the greedy algorithm produced by setting the temperature in the simulated annealing procedure to zero (Fig. 3F). The greedy algorithm thus prohibited transient decreases in the objective function during optimization. For comparison, we used hemispheres of P5 and P6 brains, where the P5 brain was preliminarily registered to a P4 → … → P0 reference brain. This is an extreme example intended for illustration purposes; in our algorithm, we register brains in the order that makes differences between brains less pronounced (see below). We show that the greedy algorithm did not succeed in registering dissimilar brain samples (e.g. mismatch in the hippocampus in Fig. 3F), whereas our attention-gated simulated annealing algorithm yielded sufficient overlap between fine brain structures (Fig. 3I). We further show that our attention mechanism, choosing the grid nodes for adjustment based on dissimilarity in their neighboring cells of the grid, improved the algorithm’s convergence. In the example where all grid nodes were selected with equal probability (no attention; Fig. 3H), the algorithm did not converge to satisfactory alignment given the same number of iterations. Finally, we show that discounting the objective function with deformation energy was also important for algorithm convergence. When undiscounted, simulated annealing deviated from the optimal solution significantly, resulting in pronounced mismatches of the brain structures (e.g. hippocampus in Fig. 3G). These observations suggest that using simulated annealing, attention-gating, and discounting the objective function with deformation improves the registration of 3D images of the brain.

We then tested the robustness and convergence rate of simulated annealing in the brain registration task. We performed registration of 28 whole mount perinatal mouse brain samples (P0–P9), sampled daily, stained against dividing cells, and pre-filtered as described in the section above (Fig. 2D). We split the brains into left and right hemispheres, registered the hemispheres from the same brain to each other, and registered pairs of right hemispheres from different brains. To save computing time and to eliminate the need to align dissimilar samples, we did not register all pairs of right hemispheres but only selected samples from the nearest time points (see “Methods” for rules of pair formation and registration order). We verified the alignment using the full collection of virtual slices. The match of fine brain structures—including cerebellum layers and the RMS—was observed in 27 out of 28 hemispheres aligned to their counterparts from the same brain (Fig. 4, top half) and then in 26 of 26 right hemispheres registered between sequential time points (Fig. 4, bottom half). Overall, our results indicate that the proposed algorithm is robust to variability in samples including diverse sets of samples of perinatal brains.

### Attention-gated simulated annealing algorithm speeds up the state-of-the-art registration

To further validate our attention-gated simulated annealing approach we compared the performance of CORGI (our algorithm) to that of the state-of-the-art tools. For this comparison, we chose the software from two whole-brain analysis pipelines: ClearMAP^19^ and CUBIC^20^, based on Elastix and ANTs image registration packages respectively. All three algorithms (CORGI, ClearMAP, and CUBIC) converged in about 5 min, however, CORGI (our algorithm) used 1 CPU core and could be deployed on a laptop while ClearMAP and CUBIC were designed to be used with 32 CPU cores, requiring a computer server.

To offer a fair comparison of registration algorithms, we kept in mind that ClearMAP and CUBIC pipelines were not optimized for use with developing brains. To account for this gap, we evaluated each algorithm on pairs of similar-age brains so that the developmental differences were not strongly manifested. Namely, we sorted all 28 brain samples in our dataset by age and performed registrations between the pairs of samples that were either of the same age or neighboring in that sequence. In addition to the full execution of ClearMAP and CUBIC algorithms, to compensate for the difference in hardware throughput (1 CPU core for CORGI versus 32 cores for ClearMAP and CUBIC), we also performed registrations using the versions of ClearMAP and CUBIC in which the number of iterations was reduced by the factor of 32. We found that the performance of ClearMAP has dropped dramatically with the reduced number of iterations. Therefore, we do not report the results obtained with the reduced version of ClearMAP.

To evaluate the performance of the four algorithms (CORGI (ours), ClearMAP, CUBIC, CUBIC-reduced), we have visually inspected the aligned brain samples to check the match of the brain regions which we have identified as problematic for automated registration. These included: (i) the lateral edges of the cerebellum (CB edge); (ii) the bulk of cerebellum (CB bulk); (iii) the rostral migratory stream (RMS); (iv) the subventricular zone (SVZ); and (v) the olfactory bulbs (OB). For each of these regions, in every pair of registered brains, a trained expert made a blind *binary* decision as to whether the region was aligned with satisfactory quality. The alignment quality was considered satisfactory if most of the aligned structures’ volume has overlapped. That is, even if the layers of the cerebellum or the RMS were offset slightly, the alignment of these structures was *not* considered satisfactory. Conversely, in the 3D OBs, small deviations in region contours were allowed. For unbiased evaluation of registration quality, the registered brain samples (spanning all ages and produced by all software) were randomly ordered and anonymized. After the expert evaluation, we computed the fractions of satisfactory alignments for each algorithm and brain region of interest shown in Table 2 below.

**Table 2:** Registration quality.

	CB edge (%)	CB bulk (%)	RMS (%)	SVZ (%)	OB (%)
CORGI	71	93	86	100	86
CUBIC	75	75	100	100	86
ClearMAP	29	68	75	93	57
CUBIC reduced	32	14	68	82	46

For each algorithm, we computed a registration quality score equal to the average of individual per-region scores. The evaluation results show that CORGI (our algorithm) and CUBIC were the most accurate in aligning the problematic regions (quality score 87%), exceeding the same measure for ClearMAP (quality score 64%). Meanwhile, CUBIC-reduced, the version which used the same number of steps as our algorithm, has only reached the quality score of 49%.

Overall, these results indicate that attention-gated simulated annealing performed on pre-filtered images offers the state-of-the-art spatial registration of brain samples. At the same time, the algorithm is not computationally demanding and can be deployed on a personal computer.

### Temporal brain registration helps reduce variability in developmental dynamics

Perinatal brains of the same age display differences in shapes, sizes, and developmental patterns. Such variability may obscure the underlying developmental process and needs to be compensated for by an alignment algorithm. Below we propose a way to account for this variability.

**Figure 5:**
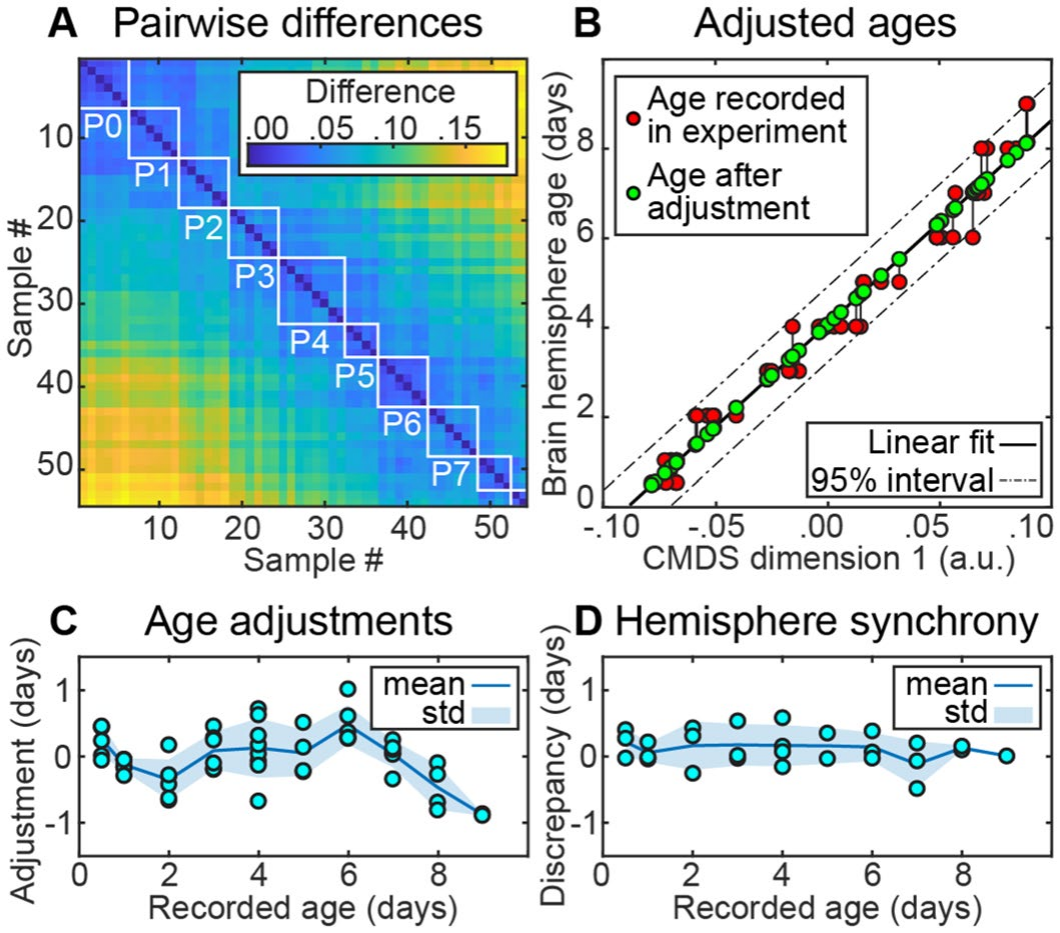
Temporal brain registration. We used differences between spatially registered brain samples to adjust the estimates of their developmental ages. (**A**) Pairwise differences (one minus Pearson correlation) between filtered 3D images of 54 hemispheres (of 27 brains). Boxes outline samples of the same ages as recorded in the experiment (P0; P1; P2 etc.). (**B**) Ages of brain samples as a function of the first CMDS component: the ages recorded in the experiment (red), linear fit (solid line) and the adjusted ages (green), 95% confidence interval (dashed line). (**C**) Age adjustment (difference between the adjusted and recorded ages) does not exceed one day. (**D**) Hemisphere synchrony (discrepancy between the adjusted ages of the left and right hemispheres in each brain).

To quantify the potential sources of variability in developing brains, we performed correlation analysis of the *filtered* brain images (mask + contours; Fig. 2D) in 27 (out of 28) well-registered samples of perinatal mouse brains (P0–P9) separated into 54 individual hemispheres. We noticed that some of the P1 brains looked like typical P0 brains; some of the P4 brains resembled P3, etc. (Fig. 5A). Similarities between a fraction of the brains of different ages implied that some variability in the brains could be explained by temporal displacements in their development. We further reasoned that significant displacements were especially likely to be observed in studies of the perinatal brain where samples are dated with respect to birth—an event only approximately related to brain development. Thus, Chuang et al.^22^ used the date with respect to conception, not birth, to define brain development stages. Overall, if temporal shifts in development underlie anatomical variability, accounting for these shifts may unmask fine details of development otherwise averaged out.

To account for temporal shifts in brain development, we implemented *temporal registration* of brain samples. We used classical multidimensional scaling (CMDS), a linear dimensionality reduction technique^18^, to “synchronize” brain samples by adjusting their ages. The CMDS algorithm placed similar brains close to each other in time and placed different ones apart based on the degree of their overall anatomical dissimilarity (Fig. 5B). Unlike nonlinear embedding techniques such as Isomap^27^, CMDS relied on both small and large-scale differences between samples. In our routine, relying only on small differences between samples for temporal registration could highlight artifacts induced by the order of spatial registration. This is because all brains within a same-age group were registered to one reference brain and imperfections of in-group registration were smaller than those across the groups. Overall, we expected temporal registration with the CMDS algorithm to resolve uncertainty in brain development pace and to uncover finer dynamics of brain development.

To test the above arguments, we performed temporal registration of 27 perinatal mouse brains (54 hemispheres). First, we show that the age adjustments using CMDS did not exceed one day and did not increase over time (Fig. 5C). The adjustments roughly corresponded to the uncertainty in the duration of mouse pregnancy^28^. At the same time, we observed no significant differences between the adjusted ages of left and right hemispheres within the same brains (Fig. 5D). The 1st CMDS dimension explained 94.5% of the variance in the embedding. These observations suggest that dating the samples relative to birth may be a major source of observed anatomical variability in the development of perinatal brains.

**Figure 6:**
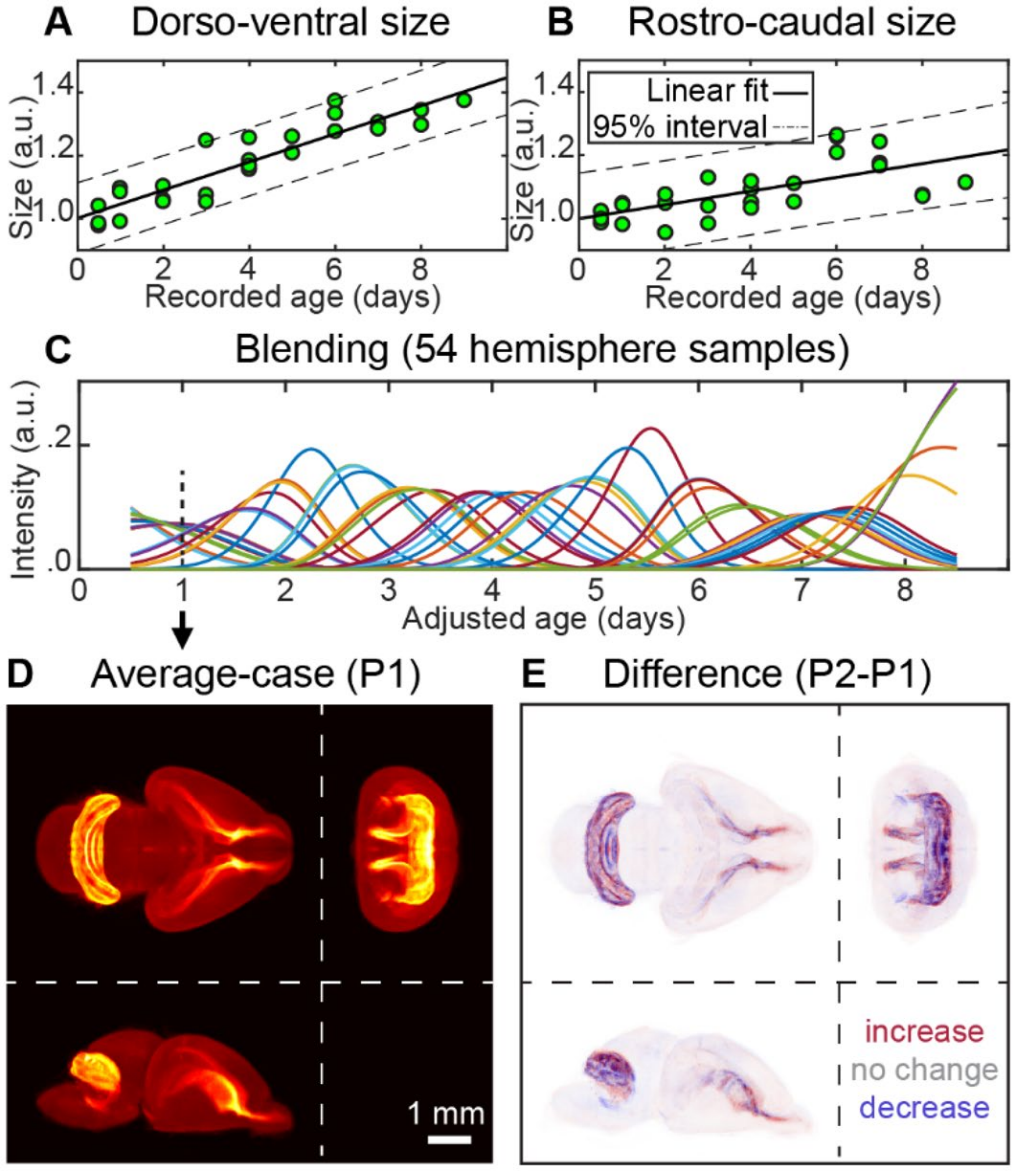
Using spatially and temporally registered brain samples to observe continuous brain development dynamics in 3D. (**A**) Dorsoventral and (**B**) rostrocaudal sizes of the brains normalized to the size at birth. Linear fit (solid line) and 95% confidence interval (dashed line). (**C**) Blending of 54 hemisphere samples. Colored lines show the weighting contributions of each hemisphere to every time point. Contribution (or intensity) is maximal at the sample’s adjusted age; it decays with a standard deviation of 1/2 of the sampling rate (1/2 day); total intensity adds up to one at every time point. (**D**) Weighted average image for P1 developing mouse brain in accordance with weighting curves (**C**). (**E**) Difference between weighted average images of P2 and P1 mouse brains. The increases and decreases in cell proliferation (EdU+ cell density) are color-coded by the intensities of red and blue respectively.

**Figure 7:**
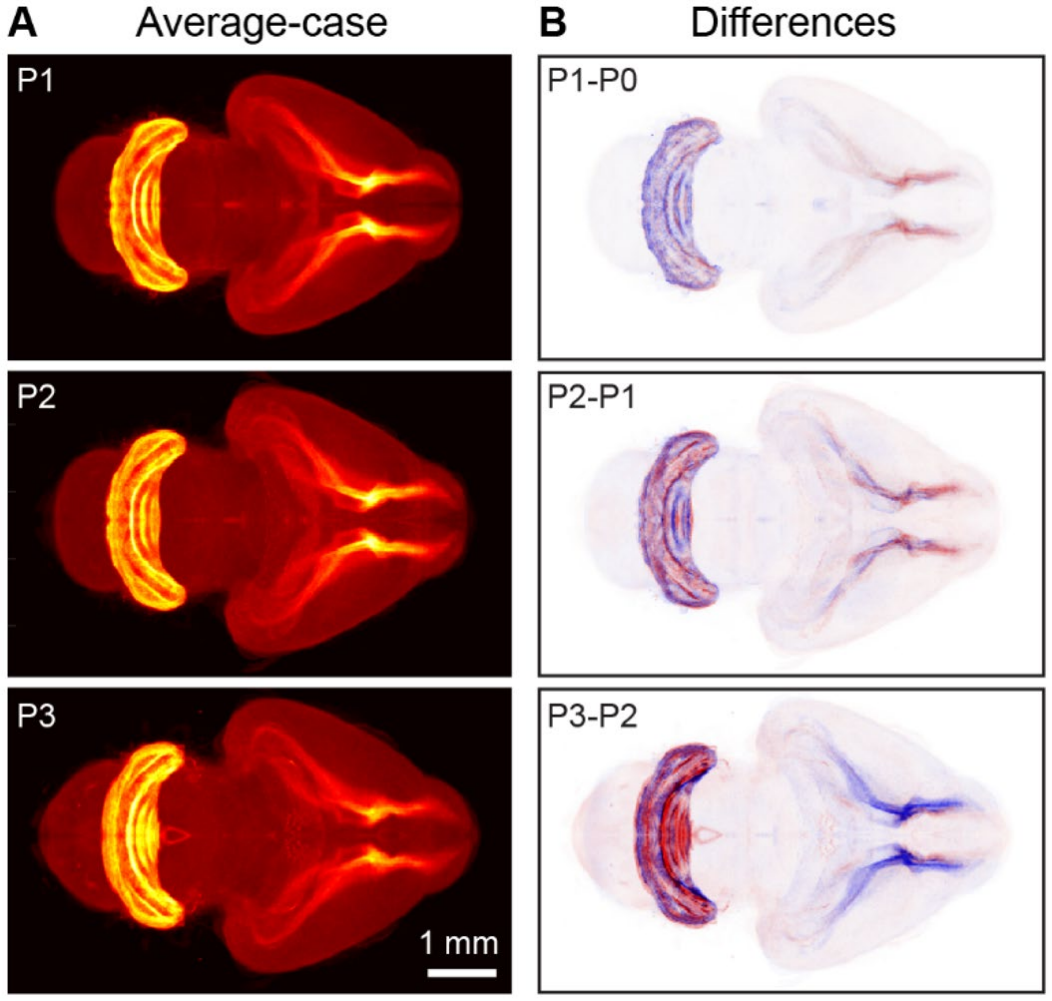
Example of continuous brain development dynamics detected using our algorithm. (**A**) Weighted average images for mouse developing brain on postnatal days P1-P3. (**B**) Average-case differences between P1-P3 and P0-P2 brains respectively reveal the dynamics of postnatal brain development. Over the course of these three days, the density of EdU+ cells in the cerebellum increases (blue to red) whereas it decreases in the RMS (red to blue). Differential images (**B**) highlight the development dynamics not easily noticeable in the weighted average images (**A**).

Determination of the samples’ developmental age allowed us to monitor developmental dynamics in an ‘average’ brain. To this end, we first distributed the registered brains on the timeline in accordance with their adjusted ages (Fig. 6C). Then we built the representation of the average brain by combining the aligned observed samples at each time point using a set of Gaussian weights. We then were able to both monitor the ongoing brain dynamics and to determine changes occurring in the distribution of EdU+ cells (Figs. 6D,E, 7). As a result, day-to-day variability in the samples (L1 norm of the daily differences over the voxels in average 3D images downsampled to 1/8 of the original resolution) decreased by 31%. This suggests that temporal shifts in development are important contributors to anatomical variability in developing brains defined on mesoscale and CMDS is efficient in estimating such shifts.

Overall, we conclude that the variability observed in perinatal brain samples may be partially explained by temporal shifts in brain development. Such shifts may arise due to a discrepancy between the moment of birth and the developmental stage of the brain at the time of analysis. These shifts can be estimated using the CMDS algorithm and—once accounted for—may reduce variability in the dynamics of brain development. We expect that data corrected this way may allow uncovering additional details of brain development dynamics.

## Discussion

In this work, we have proposed CORGI—a computational pipeline for reconstructing mesoscale dynamics of the developing mouse brain. We focused on the types of data which can only be collected ex vivo; therefore, we used multiple brains to infer the development dynamics. To combine the data, we proposed aligning 3D images of different brains (image registration). For reliable reconstruction of the development dynamics, we required high-precision alignment of variable brain samples. First, we showed that high-precision alignment can be achieved by using the contours of brain regions (Fig. 2) instead of the raw images. We then showed that contours can be efficiently aligned using simulated annealing (Fig. 3). This way, we combined the accuracy of feature-based registration approaches with the throughput of free-form approaches. We then used 28 samples of perinatal mouse brains at different developmental stages to show that our registration algorithm is robust to variability in samples (Fig. 4). Finally, we showed that individual paces of brain development can be accounted for by additionally registering brain samples in time (Fig. 5), thus smoothing (denoising) transitions between developmental stages (Fig. 6). Overall, the steps above enabled us to uncover developmental dynamics in perinatal mouse brains by using static images at different developmental stages (Fig. 7).

Reconstructing developmental dynamics from series of ex vivo samples has several advantages compared to in vivo imaging. First, ex vivo studies allow combining substantial imaging volume with high resolution. The best alternative, functional ultrasound imaging, allows to image the entire mouse brain in vivo at the resolution of 100 µm^29^. Alternatively, three-photon microscopy enables in vivo imaging at a cellular resolution up to the depth of 1300 µm^30^. For multi-day imaging typical for developmental studies, both functional ultrasound imaging and three-photon imaging may require image alignment. At the same time, ex vivo brain samples allow obtaining cellular resolution in the entire brain^22^. Using ex vivo imaging also enables the broader choice of reporter molecules, such as various fluorescent labels^2^.

CORGI is robust to potential inaccuracies of the individual algorithms used. Although each subroutine of CORGI improved registration quality, together, these algorithms play redundant roles. For example, if raw images are used for registration instead of the filtered ones, the low-variance regions such as V-SVZ-RMS may still be aligned well because of the attention mechanism in simulated annealing. Should the brain area contours be highlighted too much compared to the background, the algorithm may not get trapped in an erroneous local maximum of similarity because of temperature in simulated annealing—which allows a transient decrease in the similarity between samples. Overall, we argue that the steps of our algorithm, when combined, lead to robust registration of brain samples.

Conventionally, the scope of registration was limited to pairs of samples^31^. In this work, we have proposed a procedure for multi-sample registration, in both space and time. Our procedure allows one to use separate brain samples to reconstruct continuous dynamics of developmental processes and to trace related long-term changes (Fig. 7). The ability to capture developmental dynamics based on static data snapshots is especially important when the data can be only collected ex vivo. At the same time, our procedure supports all conventional use cases for registration algorithms, including the direct comparison of individual samples/groups and registration to common coordinate frameworks (CCFs), e.g. the Allen CCF^10^. In particular, our procedures can be combined with cell detection software, e.g. DALMATIAN^32^ or ClearMAP^33^. For conventional applications, our procedures offer high registration quality and fast convergence rates. Finally, the procedures described in this paper are modular. Depending on the task, its parts (feature extraction, spatial registration, temporal registration, data display) can be used together, separately, or in combination with other packages of the user’s choice. The described algorithms can be downloaded at http://github.com/koulakovlab/registration.
